# Human metapneumovirus prevention in children: emerging roles of monoclonal antibodies and vaccines

**DOI:** 10.3389/fimmu.2026.1932274

**Published:** 2026-09-09

**Authors:** Susanna Esposito, Valentina Fainardi, Gaia Giorgia Arnesano, Alberto Argentiero, Nicola Principi

**Affiliations:** 1 Pediatric Clinic, University Hospital of Parma, Parma, Italy; 2 Department of Pathophysiology and Transplantation, Università degli Studi di Milano, Milan, Italy

**Keywords:** children, HMPV, human metapneumovirus, monoclonal antibodies, respiratory syncytial virus, vaccines

## Abstract

Human metapneumovirus (hMPV) is a major cause of acute lower respiratory tract infection in children, older adults, and individuals with underlying medical conditions. In pediatric populations, hMPV contributes substantially to hospitalizations, morbidity, and healthcare costs, yet no licensed vaccine or monoclonal antibody is currently available for prevention. This narrative review summarizes current evidence on emerging preventive strategies against hMPV, with particular emphasis on monoclonal antibodies (mAbs) and vaccines for children. Advances in structural virology have identified the fusion glycoprotein as the principal target of neutralizing immunity and have provided a rationale for the development of hMPV-specific and cross-reactive RSV–hMPV mAbs. Several mAbs have shown potent neutralizing activity and protection in animal models, although clinical development remains limited. Vaccine development has progressed across multiple platforms, including live-attenuated, mRNA, protein-subunit, virus-like particle, and viral-vectored vaccines. However, key challenges remain, particularly regarding immunogenicity in seronegative infants, risk of enhanced respiratory disease, durability of protection, seasonality, and optimal target populations. Current evidence suggests that hMPV prevention in children will likely require a layered strategy combining maternal immunization, active vaccination during late infancy, and targeted protection for high-risk children beyond infancy.

## 1 Introduction

Since its identification by van den Hoogen et al. in 2001 (1), human metapneumovirus (hMPV) has been increasingly recognized as an important respiratory pathogen and a substantial contributor to the global burden of acute respiratory disease. Epidemiological studies have consistently shown that hMPV is among the leading viral causes of lower respiratory tract infections (LRTIs), particularly in infants, young children, older adults, and individuals with chronic underlying conditions. In children younger than 5 years of age, hMPV accounts for approximately 5–13% of respiratory hospitalizations and was estimated to cause 11.1 million LRTI episodes, 502,000 hospitalizations, and 11,300 deaths worldwide in 2018, making it one of the most important viral causes of pediatric LRTIs in many settings (2, 3). In older adults aged ≥65 years, hMPV also represents a relevant cause of severe respiratory disease, with hospitalization rates estimated at 231 per 100,000 population, corresponding to approximately 122,000 annual admissions in the United States (4).

The clinical impact of hMPV is accompanied by a considerable economic burden. Hospitalized children with hMPV infection incur substantial healthcare costs, with median hospitalization expenses reported at USD 5,513 per admission (interquartile range, USD 3,850–9,946), and significantly higher costs among patients with chronic medical conditions (5). These findings highlight the need for effective preventive strategies, particularly for pediatric populations and other groups at increased risk of severe disease.

The development of preventive interventions against hMPV has been strongly informed by advances in respiratory syncytial virus (RSV) prevention. hMPV and RSV share important virological, structural, and immunological features, including their classification within the *Pneumoviridae* family and their dependence on the fusion glycoprotein as a major target of neutralizing immunity. The recent success of RSV vaccines and long-acting monoclonal antibodies (mAbs) has therefore provided a valuable framework for the development of analogous approaches against hMPV (6). However, important differences between hMPV and RSV in age distribution, seasonality, disease severity, and high-risk populations indicate that preventive strategies cannot simply be transferred from RSV to hMPV without careful adaptation.

The objective of this narrative review is to critically examine the current landscape of hMPV prevention, with particular emphasis on mAbs and vaccines. We summarize the available preclinical and clinical evidence, discuss the biological rationale supporting these approaches, and evaluate their potential applicability, effectiveness, and limitations in pediatric populations. In addition, we address the main challenges that remain for clinical translation and identify key priorities for future research aimed at reducing the burden of hMPV-associated respiratory disease in children. This review builds upon previous broad assessments of emerging hMPV prevention and treatment strategies but addresses a distinct translational question: how should preventive interventions against hMPV be designed and implemented specifically for children? Rather than providing a general catalogue of candidate vaccines and antibodies, we focus on the relationship between pediatric disease burden, age-specific risk, seasonality, viral antigenic characteristics, and the properties of emerging preventive products. RSV is used as a clinically relevant comparator because recent implementation of maternal vaccination and long-acting monoclonal-antibody prophylaxis provides an important framework for considering hMPV prevention.

## 2 Methods

This narrative review was conducted to summarize the current evidence on the prevention of hMPV infection, with particular emphasis on preventive strategies potentially applicable to pediatric populations. A literature search was performed using PubMed/MEDLINE, Google Scholar, ClinicalTrials.gov, and relevant public health and regulatory websites, including those of the Centers for Disease Control and Prevention and the World Health Organization. The search included studies published from the identification of hMPV in 2001 through the most recent available literature.

Search terms included combinations of the following keywords: “human metapneumovirus,” “hMPV,” “vaccine,” “monoclonal antibody,” “neutralizing antibody,” “fusion protein,” “F protein,” “prefusion F,” “respiratory syncytial virus,” “RSV,” “cross-neutralizing antibody,” “pediatric,” “children,” “infants,” “high-risk,” “immunocompromised,” “congenital heart disease,” “chronic lung disease,” “prophylaxis,” and “treatment.” Additional relevant publications were identified by screening the reference lists of selected original articles and reviews.

Priority was given to original studies addressing hMPV epidemiology, pediatric disease burden, viral structure, fusion-protein antigenicity, neutralizing antibody responses, mAb development, vaccine development, animal-model efficacy, and translational strategies for prevention. Studies on RSV mAbs and vaccines were also considered when they provided relevant biological, structural, or clinical context for hMPV prevention. Reviews, surveillance reports, public health guidance, and regulatory documents were included when useful for contextualizing disease burden, seasonality, current management, or the development of preventive interventions.

Studies were considered particularly relevant if they focused on pediatric populations, high-risk hosts, hMPV F-protein epitopes, RSV–hMPV cross-neutralization, passive immunization, vaccine platforms, or clinical and translational issues relevant to future pediatric implementation. Because this was a narrative review rather than a systematic review, no formal risk-of-bias assessment or meta-analysis was performed. Evidence was synthesized descriptively, with emphasis on biological plausibility, translational relevance, and unresolved clinical questions, including patient selection, timing of administration, seasonality, dosing strategies, and the optimal pediatric age groups for future hMPV preventive interventions.

## 3 Results

### 3.1 Similarities between respiratory syncytial virus and human metapneumovirus

RSV and hMPV are among the most important causes of severe viral respiratory infections in children. Both viruses circulate predominantly during the colder months, commonly infect young children, and are major causes of LRTIs. However, despite these similarities, they differ in important epidemiological and clinical features, including seasonality, age distribution, disease severity, and the characteristics of the populations at greatest risk.

One of the most consistent distinctions between RSV and hMPV concerns their seasonal circulation. In temperate regions, RSV activity typically begins in autumn, peaks during winter, and declines in early spring. By contrast, hMPV generally circulates later, usually emerging during winter and peaking in spring. An analysis of U.S. surveillance data collected between 2014 and 2024 showed that RSV epidemics consistently preceded hMPV epidemics by several weeks. Before the COVID-19 pandemic, hMPV activity usually began in January, peaked in late March, and extended into early June, whereas RSV activity peaked substantially earlier (7). Similar patterns have been reported in Europe and Asia, where hMPV outbreaks frequently follow RSV circulation and may persist into late spring (8). The COVID-19 pandemic substantially disrupted the usual circulation of both RSV and hMPV. Non-pharmaceutical interventions implemented during 2020–2021 markedly reduced transmission of respiratory viruses, and their subsequent relaxation was followed by atypical and, in some settings, off-season epidemics (7, 8). During the early post-pandemic period, RSV showed pronounced shifts in epidemic timing, whereas hMPV circulation also became more variable, with peaks reported outside its traditional late-winter and spring window. More recent surveillance suggests a progressive return toward pre-pandemic seasonality, although geographic variability remains. Importantly, RSV generally continues to precede hMPV within the respiratory-virus season, supporting the need to consider the partially offset circulation of the two viruses when designing combined preventive strategies (7, 8).

Recent prospective U.S. surveillance provides an important contemporary comparison of pediatric RSV and hMPV disease. In the New Vaccine Surveillance Network analysis by Goldstein et al., children hospitalized with hMPV were older than those hospitalized with RSV, with median ages of 16 and 7 months, respectively, and underlying medical conditions were more frequent among infants hospitalized with hMPV. RSV infection among children presenting to emergency departments was also associated with greater odds of hospitalization, particularly in infants younger than 6 months (9). These findings support meaningful differences in the age distribution and risk profiles of severe disease caused by the two viruses. However, these patterns should not be interpreted as defining mutually exclusive populations. Considerable age overlap exists, hMPV hospitalization occurs during infancy, and RSV also causes clinically important disease beyond the first months of life (10). Thus, the available evidence suggests a shift in the relative distribution of disease burden rather than a clear age-based separation between RSV and hMPV.

Differences in disease severity and risk profiles are also clinically relevant. RSV remains the leading cause of severe viral LRTI in early infancy and is associated with high rates of hospitalization, oxygen requirement, and healthcare utilization. In the comparative analysis by Goldstein et al. (9), children presenting to emergency departments with RSV infection had significantly higher odds of hospitalization than those with hMPV infection, particularly among infants younger than 6 months of age (adjusted odds ratio, 3.27). Conversely, severe hMPV disease is more frequently associated with underlying medical conditions. Among hospitalized infants, comorbidities were more than twice as common in hMPV-infected children as in RSV-infected children. Thus, although RSV causes substantial disease in previously healthy young infants, severe hMPV disease more often occurs in older children with chronic cardiopulmonary disorders, neuromuscular diseases, immunocompromising conditions, or other significant comorbidities (11).

These epidemiological differences indicate that preventive strategies developed for RSV cannot be directly transferred to hMPV. Future hMPV vaccines and monoclonal antibodies will likely need to target a broader pediatric age range than RSV interventions and should account for the later and more variable seasonal circulation of hMPV.

Important similarities between RSV and hMPV are evident at the virological and structural levels. These similarities suggest that some technologies successfully applied to RSV monoclonal antibody and vaccine development may also inform the development of preventive strategies against hMPV. Both RSV and hMPV are enveloped, negative-sense, single-stranded RNA viruses whose entry into host cells is mediated by the viral fusion glycoprotein, or F protein, expressed on the virion surface (12). In both viruses, the F protein exists as a metastable prefusion trimer that undergoes extensive conformational rearrangement into a highly stable postfusion form during membrane fusion and viral entry. This conserved fusion mechanism represents a critical viral vulnerability and an attractive target for preventive interventions (13).

Although RSV F and hMPV F proteins share only moderate amino acid sequence identity, they exhibit substantial structural homology. Cryo-electron microscopy and other structural studies have shown that several neutralization-sensitive antigenic regions, particularly antigenic sites III and IV, are structurally conserved between the two viruses. These shared conformational epitopes can be recognized by rare broadly neutralizing monoclonal antibodies capable of inhibiting infection by both RSV and hMPV (14).

These observations provide a strong biological rationale for applying key lessons from RSV prevention to hMPV. In RSV, stabilization of the prefusion F protein has transformed the development of highly potent monoclonal antibodies and vaccines. Similarly, the hMPV F protein has become the principal antigenic target for next-generation monoclonal antibodies, vaccine candidates, and combined RSV–hMPV immunization strategies.

Nevertheless, differences exist in the accessory surface glycoproteins and in the mechanisms by which the two viruses initiate entry. Both RSV and hMPV encode F, G, and small hydrophobic (SH) proteins (15). Thus, hMPV does not lack an SH protein; rather, RSV and hMPV SH proteins differ in sequence and in their characterized biological activities. hMPV SH has been reported to modulate membrane permeability, F-mediated fusion, and innate immune responses, although its precise contribution to viral entry and pathogenesis remains incompletely defined.

The G attachment glycoproteins are even more divergent (15). Although RSV G and hMPV G occupy analogous positions as heavily glycosylated surface proteins associated with viral attachment, they have limited sequence conservation and differ in receptor interactions and relative importance for entry. hMPV G can mediate interactions with cell-surface glycosaminoglycans, but hMPV F itself also possesses important attachment functions and interacts with cellular factors involved in entry. Consequently, the distinction between an ‘attachment protein’ and a ‘fusion protein’ is less absolute for hMPV than a simple G-mediated attachment/F-mediated fusion model would imply. The two pneumoviruses therefore share the same general F-driven membrane-fusion architecture while differing in the relative contributions of G, F, and accessory proteins to attachment and initiation of infection.

Additional differences occur at the sequence and antigenic levels within an otherwise strongly conserved F-protein structural framework. RSV and hMPV F proteins share only approximately one-third of their amino-acid sequence while retaining remarkably similar prefusion architectures (16, 17). Consequently, corresponding antigenic regions can be present in both viruses but differ substantially in the residues contacted by individual antibodies, local conformation, glycosylation, and epitope accessibility. This is particularly relevant at the prefusion apex. Site Ø-related epitopes are present on hMPV F, but the hMPV apex is more extensively glycosylated and is less immunodominant than the analogous region of RSV F (16, 17). Thus, the limited cross-reactivity of most RSV-specific mAbs should not be interpreted as resulting from wholesale absence of the corresponding epitopes in hMPV; rather, it predominantly reflects limited sequence conservation and virus-specific differences in epitope composition and accessibility.

At the molecular level, the similarities between RSV and hMPV are particularly striking for the F glycoprotein. Despite sharing only approximately one-third amino-acid sequence identity, RSV F and hMPV F adopt highly similar prefusion and postfusion architectures. Their secondary-structure organization and the overall arrangement of the F trimer are therefore substantially conserved, reflecting their shared mechanism of class-I membrane fusion. Several antigenic regions also occupy corresponding structural locations on the two proteins, and sites III, IV, and V can support cross-neutralizing antibody recognition.

However, structural correspondence does not imply antigenic identity. Antibody recognition is determined by individual contact residues, local conformational features, glycosylation, and epitope accessibility. Amino-acid divergence within structurally corresponding surfaces therefore explains why most antibodies remain virus-specific despite the overall similarity of the two F proteins. Rare antibodies such as MPE8, 25P13, RSV-199, and RM5-1 recognize sufficiently conserved structural features to neutralize both RSV and hMPV and demonstrate that cross-pneumovirus antibody recognition is possible.

The prefusion apex illustrates this distinction particularly clearly. Site Ø-related antigenic surfaces are present on both viruses, but hMPV F contains a denser glycan shield and sequence differences within the region, which alter antibody accessibility and immunodominance. Thus, differences between RSV and hMPV are better viewed as variations in the molecular composition and presentation of antigenic surfaces within a substantially conserved F-protein framework rather than as wholesale gain or loss of epitopes.

The two viruses also differ in the contribution of their other surface glycoproteins to attachment and entry. Both encode G and SH proteins, but these proteins are substantially divergent in sequence and characterized function. Together, these findings indicate that RSV and hMPV share a common structural solution for F-mediated membrane fusion while retaining virus-specific molecular features that influence attachment, antigenicity, and immune recognition. This combination of structural conservation and sequence-level divergence provides the basis both for virus-specific preventive strategies and for the less common antibodies and engineered vaccine antigens capable of targeting both pneumoviruses.

### 3.2 Monoclonal antibodies

Early studies showing that polyclonal antibodies and experimental mAbs could prevent or attenuate hMPV infection in animal models provided the first proof of concept that passive immunization might represent an effective strategy against this pathogen (18, 19). Subsequent advances in antibody discovery identified several human and humanized mAbs directed against the highly conserved F glycoprotein, which is the principal target of neutralizing immunity (16, 17, 20, 21). Among these, 54G10 was one of the first fully human monoclonal antibodies shown to broadly neutralize multiple hMPV subgroups and protect against infection *in vivo*, demonstrating that conserved F-protein epitopes could be successfully targeted for passive immunization (20).

Although substantial progress has since been made in antibody discovery, no mAb has yet entered advanced clinical development or received regulatory approval for the prevention or treatment of hMPV infection. Most candidates remain in the preclinical stage, although several have demonstrated potent neutralizing activity and protective efficacy in animal models. A recently characterized and particularly promising preclinical candidate is 4F11, a fully human mAb directed against an apical prefusion-F epitope. 4F11 was selected for detailed development because of its high neutralizing potency, activity across hMPV subgroups, low susceptibility to viral escape, and therapeutic efficacy in an experimental hamster model (21). These characteristics support 4F11 as a promising preclinical candidate for further therapeutic or prophylactic development. Unlike other hMPV site Ø antibodies, which bind with a 3:1 antibody-to-trimer stoichiometry and penetrate the glycan shield between Asn57 and Asn172, 4F11 binds vertically with a 1:1 stoichiometry and directly interacts with the Asn172 glycan. This unique glycan-dependent mode of recognition illustrates how subtle differences in epitope engagement can markedly influence antibody activity and viral specificity. The close structural correspondence between RSV F and hMPV F should not be interpreted as implying widespread antibody cross-reactivity. The two proteins adopt highly similar prefusion folds but share only approximately one-third amino-acid sequence identity. Because antibody recognition depends on the identity and spatial arrangement of individual contact residues, as well as glycosylation and local conformational features, many structurally corresponding surfaces are antigenically distinct at the sequence level. Consequently, cross-neutralization is uncommon and is generally restricted to mAbs capable of recognizing particularly conserved structural surfaces, including regions within antigenic sites III, IV, and V. MPE8, 25P13, RSV-199, and RM5-1 illustrate that such cross-reactivity is possible, but these antibodies represent specialized recognition solutions rather than the expected consequence of overall F-protein structural similarity (21).

A particularly important line of investigation has focused on mAbs that exploit the shared antigenic architecture of RSV and hMPV. MPE8 and 25P13 demonstrated that conserved structural surfaces on the F proteins of both viruses can be recognized by related antibody lineages, providing a rationale for the development of broadly protective mAbs capable of targeting both pathogens (16, 20). More recently, mAbs such as RSV-199 and RM5-1 have further supported the possibility of dual RSV–hMPV prophylaxis through recognition of conserved prefusion F epitopes (16).

At the same time, the characterization of large panels of human F-specific mAbs has shown that the humoral response to hMPV differs substantially from that to RSV. In RSV, the most potent neutralizing antibodies predominantly target the prefusion apex. Human antibody-mapping studies have identified neutralizing epitopes distributed across the hMPV F surface, including sites corresponding to established pneumovirus antigenic regions as well as lateral, membrane-distal, prefusion-specific, and quaternary/trimer-interface epitopes (22). Several antibodies directed toward the trimer interface have also been described, demonstrating that this region constitutes an additional neutralization-sensitive surface rather than a unique feature represented by a single antibody (14, 21, 23, 24). This distinction has important implications for antibody design, suggesting that hMPV-specific prophylaxis may require optimization around antigenic sites that differ from those prioritized in RSV antibody development.

Recent pediatric studies are particularly relevant for future clinical application. Potent neutralizing antibodies isolated directly from hMPV-infected children have shown prophylactic efficacy in murine models, indicating that pediatric antibody repertoires can generate protective responses against distinct hMPV F-protein epitopes. These findings also suggest that antibody clonotype composition and epitope specificity may differ between children and adults, an issue that should be considered when designing preventive strategies for pediatric populations (25).

Collectively, these studies show that several hMPV-specific and cross-reactive mAbs have been identified, targeting distinct antigenic regions of the F protein and exhibiting variable breadth, potency, and *in vivo* protective activity. Although clinical development remains at an early stage, the available evidence strongly supports continued investigation of antibody-based prevention and treatment strategies for hMPV infection. Selected hMPV-specific and RSV–hMPV cross-reactive mAbs described in the literature are summarized in **Table 1**, illustrating representative target epitopes, preclinical findings, and supporting references (14, 16, 18, 20–22).

**Table 1:** Selected monoclonal antibodies targeting hMPV or conserved RSV-hMPV fusion protein epitopes.

Monoclonal antibody	Target/epitope	Development stage	Main findings	Key reference
54G10	Conserved epitope on the hMPV fusion (F) protein	Preclinical	Broadly neutralized multiple hMPV subgroups and protected against infection in animal models; provided early proof of concept for passive immunization against hMPV.	(14)
MPE8	Conserved RSV-hMPV F-protein epitope	Preclinical	Potently cross-neutralized RSV and hMPV; demonstrated the feasibility of dual pneumovirus prophylaxis using a broadly reactive monoclonal antibody.	(20)
25P13	Shared structural epitope on RSV and hMPV F proteins	Preclinical	Helped define the structural basis of RSV-hMPV cross-neutralization and identified conserved F-protein surfaces that may be exploited for broadly protective antibodies.	(16)
RSV-199	Cross-reactive prefusion F-protein epitope	Preclinical	Showed broad neutralizing activity against RSV and hMPV, supporting the development of dual-virus immunoprophylaxis strategies.	(18)
RM5-1 and related antibodies	Conserved pneumovirus F-protein epitopes	Preclinical	Demonstrated broad activity against RSV and hMPV and supported the concept of antibody strategies targeting conserved pneumovirus epitopes.	(18)
Human F-specific antibody panel	Multiple hMPV F-protein epitopes, including lateral, membrane-distal, and trimer-interface regions	Preclinical	Identified several potent neutralizing antibodies, including MPV467 and related clones; three antibodies provided robust protection in animal models and revealed hMPV antigenic sites distinct from those of RSV.	(21)
Pediatric-derived monoclonal antibodies	Multiple hMPV F-protein epitopes	Preclinical	Isolated from hMPV-infected children; all demonstrated neutralizing activity, and several showed prophylactic efficacy in murine models, highlighting the relevance of pediatric antibody repertoires.	(22)
4F11	Prefusion hMPV F-protein apex, antigenic site O	Advanced preclinical	Broadly neutralized all major hMPV lineages, showed low resistance potential, significantly reduced pulmonary and nasal viral loads in hamsters, and demonstrated therapeutic activity *in vivo*; currently one of the most advanced hMPV-specific candidates.	(22)
MPV467	Prefusion-specific hMPV F epitope	Preclinical	Potently neutralizing hMPV; demonstrated protective activity in conventional animal models and was subsequently evaluated in immunocompromised cotton rats. Both prophylactic and therapeutic administration reduced viral replication in the upper and lower respiratory tract, and therapeutic treatment reduced pulmonary pathology and inflammatory chemokine expression.	(21)

hMPV, human metapneumovirus; RSV, respiratory syncytial virus; F protein, fusion glycoprotein; mAb, monoclonal antibody.

#### 3.2.1 Potential use of hMPV monoclonal antibodies

Studies evaluating RSV prevention have shown that universal administration of long-acting mAbs to infants before their first RSV season, with additional doses for selected high-risk children during subsequent seasons, can substantially reduce RSV-related hospitalizations and generate important clinical, social, and economic benefits. However, direct extrapolation of this model to hMPV may be inappropriate because of the epidemiological and clinical differences between the two viruses.

Compared with RSV, hMPV often circulates later in the respiratory virus season and may persist for several additional weeks (26–30). Moreover, severe hMPV disease frequently occurs in children who are older than those at greatest risk for severe RSV infection and is more strongly associated with underlying medical conditions (2, 26, 28, 31). Therefore, a universal infant-only strategy analogous to current RSV mAb prophylaxis would be relatively simple to implement but could fail to protect a substantial proportion of children at highest risk of severe hMPV disease.

A more biologically and clinically plausible approach may therefore be a targeted prophylaxis program focused on high-risk children, beginning in infancy and extending through the first years of life, with repeated seasonal administration when indicated (27, 28, 32–34). Potential candidates include extremely preterm infants; children with chronic lung disease of prematurity; those with hemodynamically significant congenital heart disease; children with severe neuromuscular disorders, impaired airway clearance, or major congenital anomalies; patients with primary or secondary immunodeficiency; hematopoietic stem-cell or solid-organ transplant recipients; children with malignancies; and those with technology dependence or other forms of severe medical complexity (**Table 2**) (2, 7–9, 14, 21, 24, 27, 29, 30).

**Table 2:** Pediatric populations reported to have increased risk of severe hMPV disease and priority evidence gaps relevant to future preventive strategies.

Domain	Key evidence gap	Relevance to target population/product strategy
Disease burden	Contemporary age-specific incidence of hMPV hospitalization, ICU admission, respiratory support, length of stay, and mortality, ideally using systematic molecular surveillance.	Defines the magnitude of medical need and the proportion of severe disease potentially preventable by age-based interventions.
Risk stratification	Absolute and relative risk of severe hMPV disease in premature infants and children with chronic lung disease, congenital heart disease, neuromuscular disorders, immunocompromising conditions, transplantation, malignancy, or other medical complexity (2, 14, 21, 24, 27, 29, 30).	Identifies populations in whom risk-based prophylaxis or vaccination may provide the greatest clinical benefit and informs trial-enrichment criteria.
Infant age overlap with RSV	Granular estimates for narrow age bands, particularly 0-2, 3-5, 6-11, and 12-23 months, including the proportion of severe hMPV disease occurring during the period currently targeted by RSV prevention (9).	Determines the extent of overlap with RSV target populations and the potential value of combined RSV-hMPV products.
Post-pandemic epidemiology	Current timing, duration, magnitude, and geographic variability of hMPV circulation after disruption of respiratory-virus seasonality during the COVID-19 pandemic (7, 8).	Informs seasonal timing and whether a single administration can cover the period of greatest risk.
Post-RSV-prevention epidemiology	Absolute and proportional hMPV disease burden after widespread implementation of nirsevimab and maternal RSV vaccination.	Determines whether hMPV represents an increasing share of residual pediatric respiratory hospitalizations and whether alignment with RSV delivery remains appropriate.
Serostatus and primary infection	Age at primary hMPV infection, prevalence of baseline neutralizing antibodies, persistence of maternally derived antibodies, and relationship between serostatus and disease severity.	Informs vaccine platform selection, age at active immunization, trial design, and interpretation of immunogenicity in seronegative versus previously exposed children.
mAb target product profile	Required neutralizing potency and breadth, resistance barrier, half-life, duration of protection, pediatric dose, route of administration, safety, and ability to cover the hMPV season.	Defines whether an hMPV-specific or dual RSV-hMPV mAb is feasible for universal, age-based, or risk-based prophylaxis.
Vaccine target product profile	Safety in seronegative infants, risk of enhanced respiratory disease, number of doses, durability, breadth across hMPV lineages, mucosal/systemic immunity, and compatibility with routine pediatric vaccination schedules.	Defines the optimal age group and platform for active vaccination and whether vaccination should begin in infancy or after likely natural exposure.
Economic evidence	Direct and indirect costs of hMPV disease and cost-effectiveness of universal infant prophylaxis, high-risk prophylaxis, active vaccination, maternal immunization, and combined RSV-hMPV strategies.	Supports prioritization of target populations and informs future reimbursement and policy recommendations.
Implementation and policy	Feasibility of seasonal versus year-round administration, integration with existing immunization visits, surveillance requirements, and post-implementation effectiveness monitoring.	Translates disease-burden and product-performance data into practical recommendations and allows policy to adapt as epidemiology changes.

Risk-group categories are based on published observational studies describing increased frequency or severity of hMPV-associated disease among children with prematurity, chronic cardiopulmonary disease, neuromuscular disorders, immunocompromising conditions, transplantation, malignancy, or other major medical complexity (2, 14, 21, 24, 27, 29, 30). These categories should not be interpreted as proposed eligibility criteria for future prophylaxis. Rather, they identify populations for whom additional age-specific incidence, severity, attributable-risk, safety, and cost-effectiveness data are required before preventive recommendations can be defined. hMPV, human metapneumovirus; RSV, respiratory syncytial virus; mAb, monoclonal antibody; ICU, intensive care unit.

Nevertheless, the optimal age range, timing of administration, dosing schedule, and eligibility criteria for hMPV mAb prophylaxis remain undefined. Dedicated studies will be required to quantify disease burden in specific risk groups and to evaluate pharmacokinetics, safety, duration of protection, implementation feasibility, and cost-effectiveness in pediatric populations.

#### 3.2.2 Dual RSV-hMPV monoclonal antibodies

The introduction of dual RSV–hMPV mAbs into clinical practice could simplify prevention of the two human pneumoviruses that constitute major causes of pediatric lower respiratory tract disease. A single administration capable of protecting against both pathogens could streamline immunization schedules, reduce healthcare utilization and administration costs, and potentially improve parental acceptance and adherence. However, several scientific, clinical, and economic challenges must be addressed before this approach can be considered viable.

One major concern is that cross-reactivity may come at the expense of neutralizing potency. To achieve activity against both RSV and hMPV, dual mAbs must target conserved epitopes shared by the F proteins of the two viruses. However, these conserved regions are not necessarily the most vulnerable or immunodominant neutralization sites on either pathogen. As a result, cross-reactive antibodies may bind with lower affinity or inhibit viral fusion less efficiently than mAbs optimized for a single virus. The broadly neutralizing mAb MPE8 is a representative example: it recognizes a conserved RSV–hMPV F-protein surface and neutralizes both viruses. However, cross-reactive antibodies must accommodate amino-acid differences between the two F proteins, whereas virus-specific antibodies can exploit pathogen-specific contact residues that may permit greater binding affinity or neutralization potency (20). Thus, broad cross-reactivity does not necessarily imply reduced potency, but maintaining high neutralizing activity against two sequence-divergent viruses represents an additional constraint for mAb development.

Clinical and epidemiological considerations are equally important. In healthy infants, the added value of hMPV coverage may be limited because the burden of severe RSV disease is substantially greater than that of hMPV disease in early infancy (3, 9). Because dual mAbs would likely be more complex and costly to develop, manufacture, and evaluate than pathogen-specific products, the incremental benefit of preventing hMPV infection may not always justify the additional expenditure in universal infant programs. The greatest clinical and economic value of dual RSV–hMPV prophylaxis may therefore be expected in selected high-risk infants and young children, in whom the burden of severe disease attributable to both pathogens is higher.

The optimal age range for combined prophylaxis also remains uncertain. Dual RSV–hMPV mAbs may be most attractive during the first year of life, when RSV prevention has clear clinical relevance and hMPV prevention could provide additional benefit in vulnerable children. Beyond infancy, however, the value of combined prophylaxis may decline as the burden of severe RSV disease decreases, whereas hMPV risk may persist mainly in children with chronic or complex medical conditions. In such cases, hMPV-specific prophylaxis, rather than combined RSV–hMPV protection, may prove more appropriate.

Finally, durability of protection remains unresolved. Long-acting RSV mAbs such as nirsevimab achieve season-long protection through Fc engineering (35). Whether broadly cross-reactive RSV–hMPV mAbs, such as MPE8, can maintain sufficient serum concentrations and neutralizing activity against both viruses throughout an entire respiratory season is unknown. This question is particularly relevant because hMPV circulation often occurs later than RSV circulation and may extend further into spring.

Overall, combined RSV–hMPV mAb preparations are scientifically attractive and could offer practical advantages, particularly for selected high-risk pediatric populations. However, their clinical role will depend on careful evaluation of neutralizing potency, duration of protection, target populations, seasonal timing, safety, and cost-effectiveness before they can be considered for routine use in infants and children.

### 3.3 Vaccines

The development of hMPV vaccines initially followed a trajectory similar to that of RSV vaccines, with early efforts focused on formalin-inactivated whole-virus preparations. However, this approach was abandoned after studies in cotton rats showed that formalin-inactivated hMPV (FI-hMPV) vaccination, although protective against viral replication, led to enhanced pulmonary disease after challenge with wild-type virus. This response was characterized by severe interstitial pneumonitis, alveolitis, and exaggerated inflammatory inflammation (36). These findings closely paralleled the historical failure of the formalin-inactivated RSV vaccine, which caused enhanced respiratory disease (ERD) in vaccinated infants during subsequent natural RSV infection, resulting in high hospitalization rates and two deaths (37). The underlying mechanism is thought to involve the induction of low-avidity, poorly neutralizing antibodies together with an imbalanced immune response characterized by Th2 polarization and exaggerated pulmonary inflammation (38).

Recognition of vaccine-enhanced disease in both RSV and hMPV redirected vaccine development toward platforms capable of inducing potent neutralizing antibodies and balanced cellular immunity. These include live-attenuated vaccines (LAVs), mRNA vaccines, protein-based vaccines, virus-like particle (VLP) vaccines, and viral-vectored vaccines (3). Because RSV and hMPV together account for a substantial proportion of severe viral lower respiratory tract infections in childhood, considerable interest has also focused on bivalent RSV–hMPV vaccines. The rationale is compelling: a single vaccine could potentially prevent two of the most important causes of pediatric viral bronchiolitis and pneumonia. However, no combined RSV–hMPV vaccine has yet progressed beyond early clinical development. Important questions remain regarding immunogenicity in seronegative infants, the risk of ERD, potential interference between vaccine antigens, durability of protection, and cost-effectiveness. Consequently, substantial additional preclinical and clinical work will be required before combined RSV–hMPV vaccination can become a realistic strategy for pediatric prevention. The principal hMPV vaccine platforms and candidates currently described in preclinical or clinical studies are summarized in **Table 3**, including their antigenic targets, development stage, main findings, and key supporting references (36–56).

**Table 3:** Human metapneumovirus vaccine candidates and platforms under preclinical or clinical development.

Vaccine platform/candidate	Antigen or construct	Development stage	Main findings	Key references
Formalin-inactivated hMPV vaccine	Whole-virus formalin-inactivated hMPV	Abandoned; preclinical	Reduced viral replication after challenge but caused vaccine-enhanced pulmonary disease in cotton rats, with interstitial pneumonitis, alveolitis, and exaggerated inflammatory responses. This mirrored the historical experience with formalin-inactivated RSV vaccines and led to discontinuation of this approach.	(36–39)
Live-attenuated hMPV vaccines with gene deletions	Recombinant hMPV lacking nonessential genes such as SH, G, or M2-2	Preclinical	Attenuated replication while preserving immunogenicity and protection in animal models; supports the feasibility of live-attenuated hMPV vaccine development.	(40, 41)
Temperature-sensitive live-attenuated hMPV vaccines	hMPV candidates incorporating genetically stabilized attenuation mutations derived from RSV vaccine development, including Δ1238 and C1238S/Y1246K	Preclinical	Attenuated, genetically stable, immunogenic, and protective against hMPV challenge in hamster models; suitable for further clinical evaluation.	(42)
rhMPV-Pa	Recombinant chimeric hMPV expressing the P protein from avian metapneumovirus subtype C	Phase I clinical trial	Administered intranasally to adults, hMPV-seropositive children, and hMPV-seronegative infants and children. It showed acceptable safety and restricted replication in adults and seropositive children but was overattenuated and poorly immunogenic in seronegative children.	(43, 44)
mRNA-1653	Bivalent mRNA vaccine encoding hMPV and parainfluenza virus type 3 fusion proteins	Phase I/phase Ib clinical trials	Well tolerated in adults and seropositive children. Induced neutralizing antibodies against hMPV and PIV3, with limited additional boosting after a second dose. Safety and efficacy in seronegative infants remain unknown.	(45–47)
Stabilized prefusion F protein subunit vaccines	Stabilized hMPV prefusion F immunogens	Preclinical	Elicited potent neutralizing antibody responses and protection against viral challenge in animal models, including mice, cotton rats, and nonhuman primates; supports prefusion F as a preferred vaccine antigen.	(48, 50)
PIV3-vectored hMPV F vaccine	Parainfluenza virus type 3 vector expressing hMPV F protein	Preclinical	Induced humoral and cellular immune responses and protected African green monkeys against wild-type hMPV challenge.	(49)
VXB-241	Bivalent RSV-hMPV prefusion F protein vaccine	Phase I clinical evaluation in older adults	Showed clinically acceptable safety and reactogenicity and induced strong neutralizing immune responses against RSV and hMPV. RSV responses were comparable to those induced by a licensed adjuvanted RSVPreF3 comparator, suggesting no major antigenic interference. Pediatric data are not yet available.	(51, 56)
Experimental hMPV VLP vaccines	Virus-like particles incorporating hMPV F and M proteins or hMPV F and G glycoproteins	Preclinical	Induced neutralizing antibody and T-cell responses, reduced lung viral loads, and protected against hMPV challenge without evidence of vaccine-enhanced disease in animal models.	(52, 53)
IVX-A12	Bivalent RSV-hMPV VLP vaccine displaying prefusion F proteins from both viruses on separate VLPs	Phase I/II clinical trials in older adults	Demonstrated favorable safety and immunogenicity. Increased RSV- and hMPV-neutralizing antibodies, with responses maintained for up to 12 months in phase I and confirmed tolerability and immunogenicity in phase II studies. Pediatric efficacy remains unknown.	(54, 55)
Viral-vectored hMPV vaccines	Adenoviral or bovine/human PIV3-based vectors expressing hMPV F protein	Preclinical/early clinical development	Generated mucosal and systemic immune responses and protective efficacy in animal models. Early clinical evaluation has begun for some intranasal candidates, but data in seronegative infants remain limited.	(49)

hMPV, human metapneumovirus; RSV, respiratory syncytial virus; F protein, fusion glycoprotein; pre-F, prefusion F protein; LAV, live-attenuated vaccine; PIV3, parainfluenza virus type 3; VLP, virus-like particle.

#### 3.3.1 Live-attenuated vaccines

Although LAVs are unsuitable for immunocompromised individuals and carry a theoretical risk of reversion to virulence, they have traditionally been considered one of the most attractive approaches for infant immunization. LAVs mimic natural infection and can induce broad mucosal, humoral, and cellular immune responses. Moreover, unlike protein-based vaccines, they may be less affected by maternally derived antibodies and can stimulate local immunity in the upper respiratory tract, potentially reducing both disease severity and viral transmission (39).

Reverse-genetics studies have shown that deletion of nonessential genes, including SH, G, or M2-2, can generate attenuated hMPV strains that retain sufficient replication capacity to elicit protective immunity while maintaining an acceptable safety profile in animal models (40, 41). Another approach involves temperature-sensitive LAVs. A recent study introduced into hMPV the same genetically stabilized attenuation mutations previously used in RSV vaccine development. The resulting candidates, including Δ1238 and C1238S/Y1246K, were attenuated, genetically stable, immunogenic, and protective against hMPV challenge in hamster models, supporting their further clinical development (42).

The most advanced LAV candidate evaluated in humans is the recombinant chimeric vaccine rhMPV-Pa, which expresses the P protein from avian metapneumovirus subtype C while retaining all other proteins from hMPV (39). In a phase I clinical trial, rhMPV-Pa was administered intranasally to healthy adults, hMPV-seropositive children aged 12–59 months, and hMPV-seronegative infants and children aged 6–59 months. The vaccine showed an acceptable safety profile and was appropriately restricted in replication among adults and seropositive children. However, it proved overattenuated in seronegative children, the group most vulnerable to severe disease, resulting in limited viral replication and inadequate immunogenicity (44). These findings underscore one of the major challenges in pediatric hMPV vaccine development: achieving an appropriate balance between attenuation and immunogenicity in immunologically naïve infants.

#### 3.3.2 mRNA vaccines

The success of mRNA platforms against SARS-CoV-2 and RSV has stimulated considerable interest in mRNA vaccines for hMPV prevention. These vaccines offer several advantages, including rapid development, efficient antigen expression, and the ability to encode stabilized prefusion F protein antigens (45). The most advanced candidate is Moderna’s mRNA-1653, a bivalent vaccine encoding the fusion proteins of hMPV and parainfluenza virus type 3 (PIV3). It was initially evaluated in a phase I study enrolling healthy adults and showed an acceptable safety profile, as well as the ability to induce neutralizing antibody titers against both hMPV and PIV3 after a single dose, with limited additional boosting after a second dose (46).

In a pediatric phase Ib trial involving 27 children aged 18–55 months, mRNA-1653 was well tolerated and induced significant increases in hMPV-neutralizing antibody titers. Geometric mean fold rises ranged from 2.9 to 6.1 for hMPV-A and from 6.2 to 13.2 for hMPV-B, indicating robust immunogenicity after a single dose. The second dose provided little additional boosting, suggesting that one administration may be sufficient in previously exposed individuals (47). Despite these encouraging findings, the pediatric study population consisted exclusively of seropositive children. Consequently, the safety, immunogenicity, and efficacy of mRNA-1653 in seronegative infants, the group most vulnerable to severe primary disease, remain unknown.

In addition to mRNA-1653, Moderna has developed mRNA-1365, an investigational combined RSV–hMPV vaccine targeting both RSV and hMPV. mRNA-1365 has been evaluated in a phase I randomized, observer-blind, age-de-escalation study enrolling children aged 5 months to <24 months (53). As of May 2026, Moderna continued to list mRNA-1365 as a phase I RSV–hMPV vaccine program (54). These studies illustrate the feasibility of incorporating antigens targeting both pneumoviruses within an mRNA-based vaccination strategy, although its safety, immunogenicity, and potential clinical role in young children remain under investigation.

#### 3.3.3 Protein subunit and virus-like particle vaccines

Protein subunit vaccines targeting the hMPV F protein have emerged as one of the most promising approaches for hMPV prevention. Early studies demonstrated that stabilized prefusion F immunogens elicited potent neutralizing antibody responses and conferred robust protection against viral challenge in multiple animal models, including mice, cotton rats, and nonhuman primates (48). These findings support the selection of prefusion F as the preferred antigen for hMPV vaccine development. A PIV3-vectored vaccine expressing hMPV F induced robust humoral and cellular immune responses and protected African green monkeys against wild-type hMPV challenge (45), while more recent stabilized prefusion F subunit vaccines elicited high neutralizing antibody titers in rhesus macaques (50).

Several combination RSV–hMPV protein-based vaccines incorporating prefusion hMPV F antigens have entered human clinical trials, including the bivalent prefusion F RSV–hMPV vaccine VXB-241. Consistent with encouraging preclinical results (51), an interim analysis of a phase I study showed that VXB-241 had a clinically acceptable safety and reactogenicity profile and elicited strong neutralizing immune responses against both RSV and hMPV in older adults. Neutralizing immune responses against RSV-A and RSV-B induced by VXB-241 were at least comparable to those obtained with the licensed adjuvanted RSVPreF3 active comparator, suggesting no meaningful antigenic interference between the two viral components. However, these studies have so far been conducted only in adults. Their ability to induce protective immunity in infants, especially seronegative infants, remains unknown. In addition, concerns regarding ERD continue to influence safety assessment and clinical development strategies.

VLP vaccines mimic native viral structures but lack replication capacity, offering a potentially safe and immunogenic platform for hMPV vaccine development. Early experimental VLPs incorporating the hMPV F and M proteins induced both neutralizing antibody and T-cell responses in mice and provided complete protection against pulmonary viral replication without evidence of vaccine-enhanced disease (55). Similarly, VLPs displaying the hMPV F and G glycoproteins generated broad cross-neutralizing immunity and significantly reduced lung viral loads following challenge with heterologous hMPV strains (56).

Building on these promising preclinical findings, VLP-based candidates have progressed into human clinical development. The most advanced VLP vaccine currently under evaluation is IVX-A12, a bivalent RSV–hMPV vaccine containing prefusion F proteins from both viruses displayed on separate VLPs. In a phase I trial involving adults aged 60–75 years, IVX-A12 demonstrated a favorable safety profile and induced up to 4-fold increases in RSV-neutralizing antibodies and up to 5-fold increases in hMPV-neutralizing antibodies, with responses maintained for at least 12 months after vaccination (56). Subsequent phase II studies confirmed its immunogenicity and tolerability (60). In a group of 264 participants, solicited adverse reactions were mild to moderate in severity and were reported by 57.3% of recipients of unadjuvanted IVX-A12, 75.0% of recipients of IVX-A12 plus MF59^®^, and 37.7% of placebo recipients. From baseline to day 28, RSV-A and RSV-B antibody levels increased approximately 5–6-fold and 3–4-fold, respectively, while neutralizing antibodies against hMPV-A and hMPV-B increased approximately 2–3-fold. At day 365, antibody levels in IVX-A12 recipients remained above baseline for all responses except hMPV-A neutralizing antibodies and were higher than those observed in placebo recipients. The addition of MF59^®^ did not substantially enhance antibody responses or prolong their duration (57).

Similarly, the recombinant prefusion RSV–hMPV protein vaccine VXB-241 demonstrated strong immunogenicity in preclinical studies and has progressed to phase I clinical evaluation in older adults following encouraging preclinical results (54). Although these findings support continued development of protein-based and VLP-based RSV–hMPV vaccines, their relevance to pediatric prevention remains uncertain until safety and immunogenicity are demonstrated in infants and young children.

A complementary protein-based strategy involves the development of chimeric pneumovirus F antigens designed to combine neutralization-sensitive regions from RSV and hMPV within a single immunogen. Early structure-guided studies demonstrated that antigenic determinants from one pneumovirus F protein could be introduced into the F-protein scaffold of the other while preserving relevant conformational epitopes and inducing antibodies capable of recognizing both viruses (58, 59). In particular, chimeric constructs incorporating selected RSV and hMPV F antigenic sites elicited cross-neutralizing antibody responses and provided protection against heterologous viral challenge in animal models, supporting the feasibility of epitope grafting as an approach to broaden pneumovirus immunity (58, 59). More recently, the RHMS-1 chimeric immunogen was developed by combining immunodominant regions of RSV F and hMPV F within a single protein construct. RHMS-1 induced neutralizing antibody responses against both viruses and protected mice against RSV and hMPV challenge, providing further proof of concept for a pan-pneumovirus vaccine strategy (60). These approaches are particularly relevant because RSV and hMPV F proteins retain substantial structural similarity despite relatively low amino-acid sequence identity; therefore, engineered chimeric antigens may allow virus-specific neutralizing determinants from both pathogens to be presented within a shared structural framework rather than relying exclusively on the relatively limited number of naturally cross-reactive epitopes. Although these candidates remain at the preclinical stage, they represent a promising strategy for the development of broadly protective RSV–hMPV protein-based vaccines.

#### 3.3.4 Viral-vectored vaccines

Viral-vectored vaccines represent another promising approach because they can induce both neutralizing antibody and T-cell responses while potentially overcoming some limitations of protein subunit vaccines. Several platforms, including adenoviral vectors and bovine/human parainfluenza virus type 3 (B/HPIV3)-based vectors expressing the hMPV F protein, have demonstrated protective efficacy in animal models and generated substantial mucosal and systemic immune responses.

Early clinical development has recently begun for some vectored candidates, particularly intranasal B/HPIV3-hMPV vaccines evaluated in seropositive children. Nevertheless, published clinical data remain limited, and no evidence is yet available regarding immunogenicity or efficacy in seronegative infants. Thus, although vectored vaccines may theoretically be better suited than subunit vaccines for inducing primary immunity, this hypothesis remains unproven in the age group most in need of protection.

## 4 Discussion

Current evidence supports the biological feasibility of mAb prophylaxis against hMPV. However, unlike RSV, available epidemiological data do not currently support universal administration to all infants using a strategy analogous to existing RSV immunoprophylaxis programs. RSV prophylaxis is primarily designed to protect infants during their first RSV season, when the burden of severe disease is greatest. In contrast, hMPV-associated hospitalization generally occurs at older ages, with hospitalized children being significantly older than those hospitalized with RSV. Severe hMPV disease also frequently occurs beyond the first year of life, particularly among children with underlying medical conditions. Therefore, administration of hMPV mAbs solely before the first respiratory season could leave a substantial proportion of susceptible children unprotected.

Consequently, the most appropriate initial application of hMPV mAbs is likely to be a targeted prophylactic strategy focused on infants and children at highest risk of severe disease. These groups may include premature infants and children with chronic lung disease, congenital heart disease, neuromuscular disorders, immunocompromising conditions, or other severe comorbidities. Whether broader or universal prophylaxis ultimately becomes justified will depend on the results of clinical efficacy studies, the duration of protection conferred by long-acting antibodies, the evolving epidemiology of hMPV in the post-RSV immunization era, and formal cost-effectiveness analyses.

The evidence required to define target populations differs between passive and active immunization. For hMPV mAbs, the central epidemiological question is whether severe disease is sufficiently concentrated within predictable age groups, seasons, or clinical-risk categories to make temporary passive protection clinically and economically efficient. High-resolution estimates of hospitalization and intensive-care-unit admission rates by month of age, underlying condition, season, and geographic region are therefore required. Particular attention should be given to children during the first year of life, because the degree of overlap between the infant RSV and hMPV burdens will determine whether combined RSV–hMPV prophylaxis could provide meaningful incremental benefit over RSV prophylaxis alone.

For vaccination, target-population definition requires additional immunological information. The age at primary hMPV infection, baseline serostatus, persistence of maternally derived antibodies, immune maturation, and safety of specific vaccine platforms in infection-naïve infants will all influence the appropriate timing of active immunization. Vaccines intended for seronegative infants will require a particularly high level of safety evidence because of the historical concern regarding vaccine-enhanced respiratory disease with non-live respiratory-virus vaccines. In contrast, protein-subunit, virus-like particle, or mRNA vaccines may have different risk-benefit profiles in previously exposed older infants, toddlers, or high-risk children.

Thus, alignment with RSV prevention should not be assumed *a priori*. Instead, the degree of overlap should be quantified empirically. If a substantial fraction of severe hMPV disease occurs during the same age window in which RSV prophylaxis is already delivered, a combined RSV–hMPV product could be attractive. Conversely, if a large proportion of clinically important hMPV disease occurs after protection against RSV is no longer routinely indicated, an hMPV-specific vaccine or extended risk-based prophylactic strategy may be more appropriate.

The epidemiological and clinical differences between RSV and hMPV also raise important questions regarding the role of combined RSV–hMPV mAb preparations. Cross-neutralizing antibodies targeting conserved pneumovirus epitopes are scientifically attractive and could simplify prevention by providing protection against both pathogens with a single product. However, the broader age distribution of severe hMPV disease and the stronger association with underlying comorbidities suggest that combined RSV–hMPV prophylaxis may not provide optimal value as a universal infant strategy. At least initially, such products may be more appropriately reserved for carefully selected high-risk pediatric populations rather than administered routinely to all infants.

Although no hMPV vaccine has yet been licensed, the characteristics of vaccine candidates currently in development provide important insights into their potential pediatric use. A major challenge is that severe hMPV disease is distributed across a broader pediatric age range than RSV and is more closely linked to chronic medical conditions. Therefore, a prevention strategy identical to current RSV programs is unlikely to be optimal. Based on current evidence, the most realistic future pediatric strategy may involve a layered approach. Maternal immunization could provide passive protection during the first months of life, whereas active immunization during late infancy, approximately 6–12 months of age, could extend protection into the period when hMPV-associated hospitalization remains substantial.

The suitability of specific vaccine platforms will likely vary according to age, immune status, and risk profile. LAVs may be particularly attractive for young children because they mimic natural infection and can induce mucosal, humoral, and cellular immunity. However, no effective live-attenuated hMPV vaccine is currently available, and achieving the appropriate balance between attenuation and immunogenicity in seronegative infants remains a major challenge. In contrast, protein-subunit, virus-like particle, and mRNA vaccines may be more suitable for older infants, toddlers, and preschool children, especially after prior natural exposure. Current clinical studies of mRNA-1653 and RSV–hMPV VLP vaccines have been performed primarily in seropositive individuals and older adults, demonstrating acceptable safety and immunogenicity but providing limited information regarding efficacy in seronegative infants.

Finally, because severe hMPV disease is common among children with chronic cardiopulmonary disease, neuromuscular disorders, immunocompromising conditions, and other complex comorbidities, future prevention programs may need to extend beyond newborns and healthy infants. Targeted booster vaccination, repeated seasonal prophylaxis, or passive immunization of high-risk children beyond infancy may be required to achieve meaningful reductions in severe pediatric hMPV disease. Overall, the future prevention of hMPV in children will probably require a differentiated strategy that integrates age, seasonality, underlying risk, immune status, and vaccine or antibody platform characteristics rather than simply replicating the RSV prevention model.

Interpretation of the pediatric hMPV disease-burden literature requires caution because the available studies differ substantially in design, population, diagnostic strategy, geography, and period of observation. Estimates derived from passive surveillance, administrative databases, or clinically ordered respiratory testing may underestimate hMPV burden because hMPV testing is less consistently performed than testing for RSV or influenza. In contrast, prospective surveillance networks using systematic molecular testing are more likely to identify infections that would otherwise remain clinically undiagnosed. Differences in hospital admission thresholds, underlying population characteristics, healthcare access, viral co-detection criteria, and definitions of severe disease further limit direct comparison between studies.

Temporal context is another important source of uncertainty. Many frequently cited pediatric hMPV burden studies were conducted before the COVID-19 pandemic, which markedly disrupted the circulation of respiratory viruses. Post-pandemic surveillance has demonstrated changes in both the timing and magnitude of hMPV activity, and recent U.S. data suggest that hMPV-associated hospitalization rates increased in several pediatric age groups after the emergence of COVID-19. Consequently, historical estimates may not fully represent the current epidemiological landscape.

Similarly, comparisons between RSV and hMPV are entering a new epidemiological period following implementation of highly effective RSV preventive interventions. Widespread use of nirsevimab and maternal RSV vaccination is expected to reduce RSV-associated disease substantially among young infants. Even if the absolute incidence of hMPV remains unchanged, its proportional contribution to medically attended respiratory disease and hospitalization may therefore increase. This changing background means that historical RSV-to-hMPV comparisons cannot necessarily be assumed to predict the future relative burden of the two pathogens.

Age-related differences should also be interpreted probabilistically rather than categorically. Although contemporary surveillance indicates that the median age of hospitalization is greater for hMPV than for RSV, substantial overlap exists between their age distributions. Infants remain an important component of the hMPV hospitalization burden, while hMPV also affects older children, particularly those with underlying medical conditions. Consequently, existing data do not yet justify a simple dichotomy in which RSV prevention is directed toward infants and hMPV prevention toward older children. More granular age-specific incidence and severity estimates are needed to determine how much the optimal target populations for the two pathogens truly overlap.

## 5 Conclusions

Current evidence suggests that pediatric hMPV prevention may ultimately require a strategy that differs in important respects from existing RSV programs, but the extent of this differentiation cannot yet be defined with confidence. Contemporary surveillance indicates that hMPV-associated hospitalization occurs at an older median age than RSV hospitalization and is more frequently associated with underlying medical conditions; however, substantial overlap exists, particularly during infancy. Moreover, post-pandemic changes in respiratory-virus circulation and widespread implementation of nirsevimab and maternal RSV vaccination are likely to alter the relative epidemiological landscape in which future hMPV interventions will be introduced.

The immediate priority is therefore not only continued development of effective hMPV mAbs and vaccines but also generation of sufficiently granular disease-burden data to define whom those products should protect, when they should be administered, and what clinical benefit they must provide. Age-specific and risk-specific surveillance, systematic hMPV testing, assessment of post-pandemic seasonality, evaluation of disease burden after RSV-prevention implementation, studies in medically vulnerable children, and health-economic analyses will be essential for constructing differentiated target product profiles.

These data will ultimately determine whether pediatric hMPV prevention is best achieved through universal infant protection, targeted prophylaxis of high-risk children, active vaccination later in infancy, combined RSV–hMPV products, or a combination of these approaches. Until such evidence is available, proposed preventive strategies should be regarded as testable frameworks rather than established policy recommendations.
